# The Relationship Between Multi-Layered Ecological Support, Intrapersonal Traits, and Secondary School Students’ Self-Esteem

**DOI:** 10.3390/bs16071132

**Published:** 2026-07-06

**Authors:** Byeolbee Um, Gerta Bardhoshi, Jihyeon Choi

**Affiliations:** 1 Department of Educational Psychology, The University of Oklahoma, 820 Van Vleet Oval, Norman, OK 73019, USA; byeolbee.um-1@ou.edu; 2 Department of Counselor Education, The University of Iowa, 210 Lindquist Center, Iowa City, IA 52242, USA; gerta-bardhoshi@uiowa.edu; 3 Seoul Metropolitan Office of Education, 27, Duteopbawi-ro, Yongsan-gu, Seoul 04335, Republic of Korea

**Keywords:** secondary school students, self-esteem, intrapersonal traits, parental support, school counseling

## Abstract

This study aimed to examine the relationship between intrapersonal and environmental variables and self-esteem among secondary school students who received school counseling services. We conducted hierarchical multiple regression analysis using national data collected from 8th-and 10th-grade students in 2024 (*N* = 1636), as a part of the Monitoring the Future research study that has collected longitudinal data since 1975. The results of hierarchical multiple regression analyses indicated that intrapersonal traits, including academic achievement, happiness, and future readiness, were significantly associated with positive/negative self-esteem in secondary school students. In addition, in terms of multi-layered environmental factors, we found that parental academic support was significantly associated with positive self-esteem in secondary school students, whereas school counseling support was significantly associated with negative self-esteem. The findings of this study provide empirical evidence on adolescents’ self-esteem development, grounded in ecological frameworks proposed in the fields of education and school counseling. Implications and recommendations for counseling researchers and school personnel are provided.

## 1. Introduction

Students develop through evolving interactions with multiple layers of their environment, not only being shaped by these contexts but also actively shaping them (Lemberger-Truelove & Bowers, 2019; Lerner et al., 2021; McMahon & Mason, 2019). An ecological perspective in psychology and education traces back to Bronfenbrenner’s (1979) ecology of human development, which explained the multi-layered systems that affect human development and how they interact with one another. Specifically, he claimed that there were four layers of systems—the microsystem, mesosystem, exosystem, and macrosystem—that surrounded an individual (Bronfenbrenner, 1979). A microsystem refers to the closest interpersonal relationships (e.g., family and school), and a mesosystem is the interconnections among microsystems (e.g., the relationship between a family and a school; McMahon & Mason, 2019). An exosystem is external settings that influence an individual’s microsystem (e.g., parents’ work environments), and a macrosystem refers to broader social forces that affect the aforementioned three systems, such as laws or cultural values (McMahon & Mason, 2019).

Based on Bronfenbrenner’s (1979) theory, counseling researchers have suggested useful frameworks to understand student development and promote effective counseling services for them. For instance, McMahon and Mason (2019) developed ecological school counseling, which emphasized school counselors’ roles in understanding students within their multiple contexts and employing multilevel interventions. The counseling field further embraced the socioecological model (McLeroy et al., 1988) utilized in public health to explain how individuals’ health outcomes are impacted by environmental factors. In turn, the Association for Multicultural Counseling and Development endorsed the Multicultural and Social Justice Counseling Competences (Ratts et al., 2016), which emphasize counselors’ roles in providing counseling and advocacy interventions across six socioecological levels: intrapersonal, interpersonal, institutional, community, public policy, and international/global. For example, in order to create multilevel changes for students from low socioeconomic backgrounds, school counselors can provide individual counseling to improve students’ self-regulation (i.e., individual level), offer small-group counseling services for students to work with peers from similar backgrounds (i.e., interpersonal level), plan professional development for teachers and school staff (i.e., institutional level), and participate in political activism to remove barriers for students from disadvantaged backgrounds (i.e., community level; McMahon & Mason, 2019).

In terms of key psychological indicators that positively affect adolescent development, self-esteem is one of the most researched concepts, influenced by various environmental challenges and sources of support. A review of existing studies on self-esteem indicated its benefit to various life domains, including school, health, work, and relationships, regardless of age, gender, or race/ethnicity (Orth & Robins, 2022). Self-esteem, which can be defined as the evaluation and conceptualization that an individual makes toward oneself, evolves as an individual experiences different stages in life (Abdel-Khalek, 2016). Adolescent self-esteem is essential for adolescents’ development, because it is linked to desirable outcomes, such as mental health, prosocial behavior, life satisfaction, cognitive performance, and academic engagement. Longitudinal studies found that high adolescent self-esteem predicted less anxiety, depression, and attention-related symptoms, and better academic engagement, cognitive performance, and prosocial behaviors in the long term (Fu et al., 2017; Henriksen et al., 2017; Wang et al., 2023; Zhao et al., 2021). Also, it was reported that higher adolescent self-esteem and greater life satisfaction were associated through the mediation of increased openness and reduced difficulty in peer communication (Szcześniak et al., 2022).

Moreover, adolescent self-esteem can serve as a protective factor, buffering the negative effects of certain factors on their well-being. It was reported that adolescent self-esteem was a protective factor against adolescent depression (Fiorilli et al., 2019) and a moderator between adolescent hostility and suicidal behavior (Choi et al., 2019). Chen et al. (2016) found that adolescent self-esteem mediated the relationship between socioeconomic status and life satisfaction. Overall, these findings indicate that self-esteem plays a key role in explaining adolescents’ holistic development. Although there are several ways to assess self-esteem, some researchers suggest considering self-esteem as a bidimensional construct, including both positive self-esteem (e.g., self-confidence) and negative self-esteem (e.g., self-deprecation; Kahng & Mowbray, 2012; Owens, 2022; Silva Santos et al., 2022), for better conceptual and assessment precision and theoretical alignment with existing models (e.g., positive psychology).

Particularly, adolescent self-esteem warrants more attention for the following reasons. First, researchers have reported mixed results about the developmental trajectory of self-esteem during adolescence (Abdel-Khalek, 2016; Kuscuoglu & Hartas, 2024; Orth et al., 2018). Specifically, Orth et al. (2018) conducted a meta-analysis of longitudinal studies on the development of self-esteem and found that while the average self-esteem level increased from age 4 to 11, it remained stable from age 11 to 15. On the other hand, Kuscuoglu and Hartas (2024) found that self-esteem declined significantly between the ages of 11 and 14. Second, some studies emphasized the importance of the adolescent period in the formation of self-esteem, as adolescents’ self-esteem development can be fostered by their environment (Abdel-Khalek, 2016). For example, researchers highlighted the importance of family environment, as they found that adolescents’ self-esteem was positively associated with mother–adolescent attachment relationships, mothers’ emotional support, and authoritative parenting, whereas authoritarian and neglectful parenting were associated with lower self-esteem (Alfieri et al., 2018; Keizer et al., 2019; Mandal et al., 2021; Pinquart & Gerke, 2019).

Previous research on the relationship between sociodemographic factors and adolescent self-esteem has yielded mixed results, particularly concerning the roles of gender, race/ethnicity, age (grade level), and school settings. While some studies observe distinct variations in self-worth based on these identities, others suggest that such differences are often moderated by cultural and social expectations rather than being inherent (Bachman et al., 2011; Erol & Orth, 2011; Fagbohun et al., 2025; Mussina & Lawrence, 2026; Orth & Robins, 2014). For example, many researchers have found that male students reported higher self-esteem than female students, whereas numerous other studies have identified no statistically significant differences between the two groups (Bachman et al., 2011; Erol & Orth, 2011; Orth & Robins, 2014). Regarding race/ethnicity, Erol and Orth (2011) identified that different racial and ethnic groups indicated significantly different self-esteem levels and developmental trajectories. In contrast, Bachman et al. (2011) observed consistently high self-esteem among adolescents regardless of race/ethnicity and suggested that observed score variations may be explained by sociocultural factors. Given these conflicting findings, it is essential to account for and control the influence of sociodemographic factors when examining adolescent self-esteem to ensure a more accurate understanding. As a whole, these complex results necessitate deeper investigations into the multi-layered determining factors of adolescents’ self-esteem.

The literature on adolescents’ self-esteem points out several variables related to adolescent self-esteem that can be placed in different layers through the lens of an ecological perspective. Firstly, there are intrapersonal-level variables across academic, emotional, and career domains. Regarding the relationship between adolescents’ academic achievement and self-esteem, studies have shown that higher academic achievement predicted higher self-esteem among adolescents (Tetzner et al., 2017; Zheng et al., 2020). However, whereas Zheng et al.’s (2020) longitudinal study found that academic achievement and self-esteem reinforce each other in the long term, the directional relationship from greater self-esteem to higher grades was not supported by Tetzner et al.’s (2017) longitudinal study, which yielded mixed results. With regard to the emotional aspects, the significant relationship between adolescents’ happiness and self-esteem has been studied in the literature (Freire & Ferreira, 2020; C. S. Lee et al., 2017; Yap et al., 2022). Though many studies have focused on the impact of self-esteem on emotional well-being, some studies have also noted that current positive emotional status can support self-esteem development (Rodrigues et al., 2022; Tan et al., 2023). In terms of the relationship between adolescents’ future readiness and self-esteem, self-esteem appears to be associated with various career-related variables (e.g., career adaptability and career maturity; Ataç et al., 2018; Heo & Kim, 2016). Specifically, a study of adolescents leaving out-of-home care found that those with higher career identity were more likely to have higher self-esteem (H. Lee & Lee, 2024), which posits that better readiness for the future can strengthen students’ positive evaluation of themselves.

In addition to the intrapersonal factors, various environmental factors are significantly related to adolescent self-esteem. One of the main sources of social support is parents. Specifically, multiple studies have implied a positive effect of parental academic support on adolescent self-esteem. A study conducted on grade 4 students revealed that parental academic support positively affected their children’s self-concept and academic achievement (Chohan & Khan, 2010). Kuscuoglu and Hartas (2024) also found that parental behavior and practices affected adolescents’ self-esteem as well as their school attitudes and self-concept. Lastly, it was reported that adolescent self-esteem and parental involvement (including parental academic support) were associated, and adolescents’ self-esteem influenced their academic performance (Moneva et al., 2020).

Regarding the school environment, researchers have also noted that school counseling can be helpful for students with low or negative self-esteem (Felicia et al., 2025; Tolan, 2023). For example, Putri and Darmayanti (2025) identified that offering group counseling with assertive training techniques improved the self-esteem of adolescents who experienced bullying. Az-Zahro et al. (2025) found that cognitive behavioral group counseling with cognitive restructuring was effective in enhancing junior high school students’ self-esteem. Brogan et al. (2020) implemented solution-focused group counseling for Latino middle school students and reported that their self-esteem and ethnic identity increased. Similarly, there have been other types of mental health support provided to students in schools, such as mental health curricula and resources. Studies have shown that various school-based interventions (e.g., preventive mental health lessons, assertive training, family psychological education therapy) improved adolescents’ self-esteem (Jenson et al., 2023; Parray & Kumar, 2017; Ramadhan et al., 2018). As such, numerous studies have examined self-esteem as a predictor, outcome, or moderator/mediator of models explaining adolescents’ holistic development and well-being. Nevertheless, there is a dearth of research applying a multi-layered framework to understand adolescents’ self-esteem development, including intrapersonal and environmental factors.

In this study, we selected individual and environmental variables relevant to secondary school students within the microsystem (Bronfenbrenner, 1979) and across three socioecological levels (i.e., intrapersonal, interpersonal, and institutional; McLeroy et al., 1988). We excluded variables from other ecological systems and socioecological levels (i.e., the mesosystem, exosystem, and macrosystem, as well as the community, public policy, and international/global levels) because the microsystem and the first three socioecological levels represent the contexts in which students have direct interactions and develop relationships. Furthermore, we confined the scope of the study to variables at or below the institutional level to derive practical and actionable implications for school personnel.

### Aim of This Study

Taken together, this study aimed to examine the relationship between intrapersonal and environmental factors and positive and negative self-esteem among secondary school students who had received school counseling services. The research questions were as follows:

1. How are intrapersonal factors (i.e., academic achievement, happiness, and future readiness) associated with positive self-esteem in secondary school students?
2. To what extent do environmental factors (i.e., parental academic support, school counseling support, and school mental health support) contribute to positive self-esteem in secondary school students, after controlling for intrapersonal factors?
3. How are intrapersonal factors associated with negative self-esteem in secondary school students?
4. To what extent do environmental factors contribute to negative self-esteem in secondary school students, after controlling for intrapersonal factors?

## 2. Materials and Methods

This study analyzed data collected from an ongoing nationwide project since 1975, Monitoring the Future (MTF). Annually, the MTF project collects survey data from adolescents across the United States, regarding their physical and psychological health and development, family and school environment, and substance use. Aligning with the aim of this study to examine secondary school students’ psychological development in consideration of multi-layered ecological support, we obtained data access permission through the ICPSR Database and selected the main variables of this study. Among the 17,351 secondary school students (8th- and 10th-graders) who participated in the 2024 MTF, due to practical and regional restrictions, the school counseling questions were not included in 7524 surveys among 17,351. Next, we excluded 690 participants who responded with “Not offered” because it does align with the response options regarding the helpfulness of counseling services. Also, we did not include 3136 participants who responded with “Don’t know/Not applicable” to questions about the helpfulness of counseling services, because this response is open to multiple interpretations; for instance, the students may not have required counseling, may have perceived themselves as ineligible, or could have been uninformed about the existence of such services. In addition, this response is also not aligned with the response options regarding the helpfulness of counseling services. Lastly, as we attempted to capture school counseling experience as a comprehensive construct of both individual counseling and group counseling, 1636 out of 6041 responses were selected for analysis. As a result, we used data from a student subgroup (*N* = 1636) who responded that they received school counseling services, both in individual and group formats.

### 2.1. Measures

#### 2.1.1. Positive Self-Esteem

Four questions were used to measure positive self-esteem in secondary school students, following the instruction: “How much do you agree or disagree with each of the following statements?”. An example item reads “I take a positive attitude toward myself”. Responses were based on a 5-point Likert-type scale, ranging from 1 (Disagree) to 5 (Agree). The total scores range from 4 to 20, with higher scores indicating more positive self-esteem. Cronbach’s α for the four items was 0.94.

#### 2.1.2. Negative Self-Esteem

We assessed negative self-esteem in secondary school students, following the instruction: “How much do you agree or disagree with each of the following statements?”. An example item reads “Sometimes I think that I am no good at all”. Responses were based on a 5-point Likert-type scale, ranging from 1 (Disagree) to 5 (Agree). The total scores range from 4 to 20, with higher scores indicating more negative self-esteem. Cronbach’s α for the four items was 0.85.

#### 2.1.3. Academic Achievement

Regarding their academic achievement, participants were asked to respond to the following question: “Which one of the following best describes your average grades in this school year?”. The available options were based on a 9-point Likert-type scale, ranging from 1 (D [69 or below]) to 9 (A [93–100]). Students’ self-reported grades are highly consistent with official academic records, supporting their use as a reliable single-item measure of academic achievement in educational research (Sticca et al., 2017).

#### 2.1.4. Happiness

A single item assessed participants’ perceived happiness, which reads “Taking all things together, how would you say things are these days–would you say you’re very happy, pretty happy, or not too happy these days?”. A 3-point Likert-type scale was used, with responses from 1 (Not too happy) to 3 (Very happy). The existing studies and international guidelines support the usefulness and appropriateness of a single-item happiness measure (Organization for Economic Cooperation and Development, 2025; Raudenská, 2023).

#### 2.1.5. Future Readiness

A single question assessed secondary school students’ readiness after school: “Which best describes your plans after secondary school?” A 4-point Likert-type scale was used, ranging from 1 (I have no idea what I will do) to 4 (I know exactly what I will do). This question captures students’ overall post-secondary preparedness, and has been widely used as a single-item indicator for educational and career aspirations in prior research (e.g., Papay et al., 2011).

#### 2.1.6. Parental Academic Support

Parental support regarding academic matters was assessed using the following question: “How often do your parents (or stepparents or guardians) do the following?”. Participants were asked to answer this question in relation to the following items: “Check on whether you have done your homework” and “Provide help with your homework when it’s needed,” based on a 4-point Likert-type scale, ranging from 1 (Never) to 4 (Often). The total scores range from 2 to 8, with higher scores indicating greater parental academic support. Cronbach’s α for the two items was 0.71.

#### 2.1.7. School Counseling Support

Participants were asked to answer the following question regarding the helpfulness of school counseling services: “During the current school year, how helpful have the following been, if provided by your school?”. Responses were provided on a 5-point Likert-type scale ranging from 1 (Not at all helpful) to 5 (Extremely helpful) for the items “Counseling support” and “Group counseling”. The total scores range from 2 to 10, with higher scores indicating greater perceived helpfulness of school counseling services. Cronbach’s α for the two items was 0.94.

#### 2.1.8. School Mental Health Support

Participants were asked to answer the following question regarding the helpfulness of school counseling services: “During the current school year, how helpful have the following been, if provided by your school?”. Responses were provided on a 5-point Likert-type scale ranging from 1 (Not at all helpful) to 5 (Extremely helpful) for the items “Mental health curriculum” and “Mental health resources”. The total scores range from 2 to 10, with higher scores indicating greater perceived helpfulness of mental health support in school. Cronbach’s α for the two items was 0.97.

#### 2.1.9. Sociodemographic Information

In terms of sociodemographic information, respondents reported their grade (1 = 8th grade and 2 = 10th grade), sex (1 = male, 2 = female, 3 = other, and 4 = prefer not to answer), race (1 = Black, 2 = White, and 3 = Hispanic), school region (1 = Northeast, 2 = Midwest, 3 = South, and 4 = West), and school setting (1 = city, 2 = suburban, and 3 = rural).

### 2.2. Data Analysis

Because Little’s missing completely at random (MCAR) test (*χ*^2^ = 459.41, *df* = 384, *p* = 0.005) did not support the MCAR of the data, the missing values (1.8%) of the data were addressed by the multiple imputation (MI) method using SPSS 25.0, with 5 imputed datasets created via the Fully Conditional Specification (FCS) method. All variables included in the main analysis were used in the imputation model. Using the data, we conducted preliminary analyses to provide means and standard deviations of the main variables and bivariate correlation coefficients between them. Before performing regression analyses, we identified normality, linearity, homoscedasticity, independence of errors, and multicollinearity of the data by examining univariate skewness (−0.40 to 0.30) and kurtosis (−1.34 to 0.14) values, histograms, Q-Q plots, variance inflation factors (VIF; 1.00 to 1.12), tolerance values (0.93 to 0.99), and Durbin–Watson statistics (1.91 to 1.98; Kline, 2016; Miles, 2005). Using G*Power 3.1.9.7, we determined that a sample size of 172 is sufficient to meet the minimum requirement for the ten independent variables included in the model, which yields a medium effect size (*f*^2^), with an α of 0.05 and a power of 0.95.

We conducted a set of analyses of variance (ANOVAs) to identify significant differences in secondary school students’ self-esteem levels according to their sex, race, grade, school region, and school setting. Subsequently, we tested our research questions using hierarchical multiple regression models including covariate(s). Three-step hierarchical regression analyses were conducted to examine positive self-esteem and negative self-esteem in secondary school students who had received school counseling services. Specifically, for each outcome variable (i.e., positive/negative self-esteem), a set of covariates were inserted as the first step, which yielded Model 1. Intrapersonal variables (i.e., academic achievement, happiness, and future readiness) were inserted as the second step, which yielded Model 2. As the third step, environmental variables (i.e., parental academic support and school counseling support) were additionally inserted on top of the covariates and intrapersonal variables, in order to examine the association with positive/negative self-esteem in secondary school students, which yielded Model 3. Finally, we conducted sensitivity analysis to confirm the robustness of our findings by substituting school counseling support with school mental health support across research models, given their high correlation coefficient.

## 3. Results

To examine the factors associated with positive and negative self-esteem, we first examined participant characteristics and conducted preliminary analyses. Subsequently, hierarchical multiple regression analyses were performed to test the proposed research questions. When considering and interpreting the results, it should be noted that the findings of this study are specific to students who have prior experience with both individual and group counseling services in school.

### 3.1. Sample Characteristics

Participants in this study included 703 students in the 8th grade (43.0%) and 933 students in the 10th grade (57.0%). In terms of sex, students self-reported as male (*n* = 762, 46.6%), female (*n* = 816, 49.9%), or other (*n* = 14, 0.9%), or selected “prefer not to answer” (*n* = 43, 2.6%). They self-reported their race as Black (*n* = 256, 15.6%), White (*n* = 699, 42.7%), or Hispanic (*n* = 306, 18.7%; race was dichotomized as White and Racial Minorities (Black and Hispanic for analysis)). We retained the missing values (*n* = 375, 22.9%) because students with other racial/ethnic identities had limited response options. Regarding school region, 26.8% were in the Northeast (*n* = 438, 26.8%), 28.8% in the Midwest (*n* = 472, 28.8%), and 44.4% in the South (*n* = 726, 44.4%). Due to restrictions on the disclosure of school-level data, data from students in schools in the West were unavailable. Participants reported their school setting as city (*n* = 406, 24.8%), suburban (*n* = 1068, 65.3%), or rural (*n* = 162, 9.9%).

### 3.2. Preliminary Analyses

Table 1 presents the descriptive statistics (i.e., means and standard deviations) and bivariate correlation coefficients between the main variables of this study. Specifically, positive self-esteem was positively associated with academic achievement, happiness, future readiness, parental academic support, school counseling support, and school mental health support (*r*s = 0.06 to 0.35). Participant negative self-esteem was negatively associated with academic achievement, happiness, future readiness, parental academic support, school counseling support, and school mental health support (*r*s = −0.06 to −0.35). Between the intrapersonal and environmental variables, most of them indicated non-significant to weak relationships (*r*s = −0.06 to 0.21), except for the strong correlation between school counseling support and school mental health support (*r* = 0.92; Cohen, 1988). These findings indicate that the self-esteem of secondary school students who received both individual and group counseling is related to their intrapersonal traits and proximal environmental support.

### 3.3. Results of Hierarchical Multiple Regression Analyses

The results of the ANOVAs indicated that sex was significantly related to both positive self-esteem (*M_Male_* = 14.07, *M_Female_* = 13.70, *F* = 3.60, *p* < 0.001) and negative self-esteem (*M_Male_* = 9.18, *M_Female_* = 11.15, *F* = 6.88, *p* < 0.001). In contrast, race, grade, school region, and school setting did not indicate significant differences in positive self-esteem (*F* values from 0.50 to 1.19, *p* values from 0.27 to 0.95) and negative self-esteem (*F* values from 0.84 to 1.28, *p* values from 0.20 to 0.64). We performed hierarchical multiple regression analyses for participant positive self-esteem and negative self-esteem, in order to examine the explanatory power of three sets of independent variables (i.e., covariates, as well as intrapersonal and proximal environmental factors). Specifically, we tested Model 1 including sociodemographic factors as covariates, with Model 2 and Model 3 representing the sequential steps of the analysis.

After testing the original models, which included covariates, three intrapersonal independent variables, and three proximal environmental independent variables, we identified a multicollinearity issue between school counseling support and school mental health support, which resulted in the exclusion of school mental health support that did not yield additional explanatory power for each outcome. As a result, school mental health support was excluded from the models, as it did not add significant explanatory value to the model for each outcome. The results of hierarchical multiple regression analyses based on the finalized research models are shown in Table 2 and Table 3.

#### 3.3.1. Positive Self-Esteem

In Model 1, five covariates were entered and sex showed a significant and negative correlation with positive self-esteem (*B* = −0.59, β = −0.10, *SE* = 0.17, *p* < 0.001), which accounted for 2% of the variance in positive self-esteem (Δ*F* = 3.75, *p* < 0.001). In Model 2, intrapersonal factors were added, resulting in a significant 16% increase in the model’s *R*^2^ value (Δ*F* = 79.12, *p* < 0.001). Specifically, academic achievement (*B* = 0.34, β = 0.19, *SE* = 0.05, *p* < 0.001), happiness (*B* = 1.92, β = 0.29, *SE* = 0.18, *p* < 0.001), and future readiness (*B* = 0.38, β = 0.13, *SE* = 0.08, *p* < 0.001) were all positively associated with positive self-esteem. Finally, in Model 3, the inclusion of environmental factors (parental academic support and school counseling support) resulted in an additional, significant 4% increase in the model’s *R*^2^ value (Δ*F* = 34.23, *p* < 0.001). Within this final model, parental academic support showed a significant positive association with positive self-esteem (*B* = 0.42, β = 0.22, *SE* = 0.05, *p* < 0.001), whereas school counseling support was not significantly associated with positive self-esteem (*B* = −0.01, β = −0.01, *SE* = 0.04, *p* = 0.81). In terms of confidence intervals, race (95% CI; [−0.218 0.592]), grade (95% CI; [−0.028, 0.357]), school region (95% CI; [−0.174, 0.297]), school setting (95% CI; [−0.130, 0.553]), and school counseling support (95% CI; [−0.081, 0.057]) included zero. The final model accounted for 22% of the total variance in positive self-esteem (*R*^2^ = 0.22), indicating a medium effect size (Cohen, 1988).

#### 3.3.2. Negative Self-Esteem

In Model 1, five covariates were entered. Sex (*B* = 1.76, β = 0.25, *SE* = 0.20, *p* < 0.001) and grade (*B* = −0.37, β = −0.08, *SE* = 0.13, *p* < 0.01) were significantly correlated with negative self-esteem, whereas race (*B* = −0.40, β = −0.04, *SE* = 0.27, *p* = 0.14), school region (*B* = 0.06, β = 0.01, *SE* = 0.16, *p* = 0.51), and school setting (*B* = −0.28, β = −0.04, *SE* = 0.22, *p* = 0.16) were not. Model 1 accounted for 7% of the variance in negative self-esteem (Δ*F* = 18.06, *p* < 0.001). In Model 2, intrapersonal factors were added, resulting in a significant 10% increase in the model’s *R*^2^ value (Δ*F* = 47.38, *p* < 0.001). Among these factors, happiness (*B* = −2.42, β = −0.31, *SE* = 0.22, *p* < 0.001) was negatively associated with negative self-esteem, whereas academic achievement (*B* = −0.08, β = −0.04, *SE* = 0.06, *p* = 0.15) and future readiness (*B* = −0.10, β = −0.03, *SE* = 0.09, *p* = 0.30) were not. Finally, in Model 3, the inclusion of environmental factors resulted in an additional, significant 5% increase in the model’s *R*^2^ value (Δ*F* = 32.69, *p* < 0.001). The final model revealed that school counseling support (*B* = −0.34, β = −0.21, *SE* = 0.04, *p* < 0.001) was significantly associated with lower negative self-esteem, whereas parental academic support (*B* = −0.10, β = −0.04, *SE* = 0.06, *p* = 0.13) was not. Compared to Model 2, academic achievement indicated significance (*B* = −0.12, β = −0.06, *SE* = 0.06, *p* < 0.05). In terms of confidence intervals, the following variables included zero: race (95% CI; [−0.057, 0.641]), school region (95% CI; [−0.254, 0.315]), school setting (95% CI; [−0.659, 0.137]), and parental support (95% CI; [−0.217, 0.029]). The final model accounted for 21% of the total variance in negative self-esteem (*R*^2^ = 0.21), indicating a medium effect size (Cohen, 1988).

To clarify the stability of our findings despite the high correlation between school counseling support and school mental health support (*r* = 0.92), we conducted a sensitivity analysis by substituting the former with the latter in our regression models. The results remained consistent across both models, with negligible differences in *R*^2^ values and no changes in the statistical significance of the primary independent variables. This stability confirms that our findings are robust and not unduly influenced by the multicollinearity between these two environmental support variables.

## 4. Discussion

The present study examined the relationship between various intrapersonal and environmental factors and the self-esteem of secondary school students who experienced both individual and group counseling, from an ecological perspective on student development (Bronfenbrenner, 1979; McMahon & Mason, 2019). This study distinguished positive dimensions of self-esteem from its negative dimensions, for precise assessment and better examination of their relationships with other psychological factors (Kahng & Mowbray, 2012; Silva Santos et al., 2022). We analyzed responses from 8th- and 10th-grade students in a national dataset (i.e., Monitoring the Future) using hierarchical regression analysis. The results indicated that intrapersonal and environmental factors were significantly associated with the self-esteem of secondary school students who received both individual and group counseling.

First, we found that three intrapersonal factors (i.e., academic achievement, happiness, and future readiness) were associated with participants’ positive/negative self-esteem across academic, emotional, and career aspects. Specifically, our study identified the relationship between adolescents’ academic achievement and self-esteem, echoing most of the relevant findings (e.g., Tetzner et al., 2017; Zheng et al., 2020). Zheng et al. (2020) explained that positive feedback following higher academic achievement improves self-perceptions of their competence, which can ultimately contribute to greater self-esteem. In addition, the results indicated that happiness had the strongest association with participants’ positive/negative self-esteem. This highlights the importance of emotional well-being in understanding individuals’ evaluation of self-worth, as existing findings explain that increased happiness boosts students’ positive perspectives of themselves, which strengthens self-esteem over time (Rodrigues et al., 2022; Tan et al., 2023). As another important developmental aspect, our findings also emphasize that future readiness is related to greater positive self-esteem, which is aligned with the findings of career-related research articles (Ataç et al., 2018; Heo & Kim, 2016; H. Lee & Lee, 2024). This suggests that students feel a sense of competence and control over their lives when they perceive a clear plan for their secondary education, which serves as a key indicator of self-esteem development (H. Lee & Lee, 2024; Pignault et al., 2023). However, we identified that their future readiness is not associated with negative self-esteem. Given the cross-sectional nature of our data, this finding suggests that future readiness may be more closely linked to the mechanisms that bolster positive self-worth rather than those that alleviate negative self-evaluations. It is possible that the cognitive benefits of career preparedness primarily function as a resource for enhancing positive self-regard, while negative self-esteem may stem from different emotional or environmental factors that function independently of career planning, which necessitates further investigation.

In addition to intrapersonal factors, we identified two environmental factors that were linked to positive/negative self-esteem. The first factor is parental academic support, specifically their involvement in monitoring and assisting with homework. The findings of this study indicate that students perceive themselves more positively when they get emotional and practical support from their parents regarding their coursework, consistent with previous studies (Chohan & Khan, 2010; Kuscuoglu & Hartas, 2024). Parental involvement in homework significantly enhances children’s self-esteem by providing emotional support, a sense of competence in learning, and psychological safety regarding failure (Abín et al., 2024; Chohan & Khan, 2010). However, we found that parental academic support was not significantly associated with negative self-esteem. This suggests that parental academic support may act primarily as a promotive factor for positive self-regard rather than as a buffer against negative self-evaluation. While parental involvement can reinforce positive self-worth by validating academic effort, negative self-esteem may be rooted in more complex emotional or environmental processes that operate independently of parental coursework assistance. These findings suggest that positive and negative self-esteem may develop through distinct psychological pathways, implying that the influence of parental support is nuanced and may vary based on the specific dimension of self-esteem in question (Givertz & Segrin, 2014; Kuscuoglu & Hartas, 2024; Marsh & O’Mara, 2008).

The other environmental factor was school counseling support, as researchers have found an effect of school-based counseling intervention on adolescents’ self-esteem (Epel et al., 2021; Felicia et al., 2025; Tolan, 2023). Regarding students’ perceptions of the helpfulness of school counseling services, Ohrt et al. (2016) reported that adolescents’ perceptions of school counseling were significantly related to various domains, including emotional/social support, therapeutic relationships, college and career readiness, life skills, advocacy, and academic achievement. Regarding covariates, sex was significantly associated with both positive and negative self-esteem, which aligns with some research suggesting that female adolescents report higher levels of negative self-esteem (e.g., Kuscuoglu & Hartas, 2024). Specifically for negative self-esteem, participant negative self-esteem was lower for 10th graders than 8th graders, consistent with existing studies indicating that older students reported higher and more stable self-esteem (e.g., Erol & Orth, 2011). In contrast, race, school region, and school setting were not significantly associated with adolescents’ self-esteem, which adds further evidence to the literature reporting mixed results regarding these sociodemographic factors. Nevertheless, it is noteworthy that both intrapersonal and environmental factors remained significantly associated with positive and negative self-esteem among secondary school students who had received both individual and group school counseling services, even after controlling for the covariates.

In this vein, our study contributes empirical evidence that individual and group counseling services that students perceive as helpful are related to improvements in their self-esteem. Notably, school counseling was only significantly associated with negative self-esteem, whereas it was not related to positive self-esteem. This may be due to limited resources, which force school counselors to prioritize urgent cases and focus primarily on symptom reduction (American School Counselor Association, 2025; Lapan et al., 2012). Students with lower self-esteem may perceive school counseling interventions as more relevant to their personal challenges and, as a result, engage more actively in an effort to improve. In contrast, students with higher self-esteem may feel less personal benefit from such interventions and, therefore, may be less likely to participate or find the counseling helpful. In addition, we excluded school mental health support due to the multicollinearity issue with school counseling support. This suggests that school counseling support and school mental health support may not be perceived as distinct entities for secondary school students, rather implying that school counseling support functions as the primary practical manifestation of mental health support that students experience. This finding is aligned with school counselors’ key role in promoting students’ mental health and school safety (American School Counselor Association, 2025). Considering the conceptual overlap, future studies assessing students’ mental health need to specify the types of services, resources, and providers when developing survey items.

Among many studies of adolescents’ self-esteem, this study focused more on the multilayered environmental factors associated with positive/negative self-esteem across individual, family, and school dimensions. Overall, each research model accounted for a meaningful proportion of variance in positive/negative self-esteem in secondary school students who experienced both individual and group counseling services in school. The findings supported McMahon and Mason’s (2019) ecological school counseling model, which emphasized the importance of systematic support for student development, in consideration of the interactions between an individual and their proximal environment. Given that this study uniquely examined the association of perceived helpfulness of school counseling and students’ self-esteem, along with intrapersonal development and family support, the findings are expected to provide useful guidelines for school counselors to design and provide systemic multilevel interventions aligned with the ASCA national model.

### 4.1. Limitations

The current research includes a few limitations. First, causal relationships between the independent variables and outcome variables should not be interpreted as evidence of causality, given that this study is cross-sectional. Another limitation of this study is the reliance on self-reported data, including academic grades. This may introduce response distortion due to social desirability bias (Podsakoff et al., 2003). While self-reporting is a common constraint in secondary datasets, our findings should be interpreted with caution. Future research could improve validity by incorporating objective institutional records. These methodological limitations should be considered when interpreting the findings, and future research would benefit from employing longitudinal designs and incorporating objective measures.

Next, this study imposes inherent limitations due to the use of secondary data. The dataset was not originally collected to address the specific research questions of this study, and we had to select variables among the existing ones. This limited the availability and scope of the data, specifically when there was only a limited number of response options. For instance, in terms of race, there were three options (i.e., White, Black, and Hispanic), and the “other” option was missing. In addition, a potential limitation of this study is the use of single-item measures from secondary data (grade, happiness, and future preparedness). While Bergkvist (2015) states that single-item measures can be valid if the target construct is sufficiently concrete and the item is clearly phrased, the findings from our regression models should be interpreted with caution, as the potential for measurement error may lead to attenuation of the estimated coefficients.

Third, the sample was limited to students who had participated in both individual and group counseling experiences in school, which excluded responses from students who had experienced only one form of school counseling service or had no access to school counseling. Given the specific characteristics of our sample, the generalizability of these findings to other contexts may be limited. Moreover, data from Western schools were not included in the MTF dataset, as they had been intentionally removed at the request of the data provider. This may impact the generalizability of this study to secondary school students in the United States.

Finally, the findings of this study may vary across subgroups, given the mixed results reported in the existing literature. For example, Vialle et al. (2015) pointed out that academic achievement was not significantly correlated with self-esteem among high-ability students. Thus, future studies can employ mixed-methods or qualitative research designs to more deeply investigate the underlying mechanisms and contextual factors that support or challenge the findings of this study.

### 4.2. Implications and Future Research

This study has several implications for researchers and school personnel, including school counselors, teachers, and administrators. First, the findings of the present study can inform school counselors of multiple strategies that are associated with secondary school students’ self-esteem. School counselors can design and develop school counseling programs aimed at fostering students’ academic, emotional, and career competencies. For example, the program can involve practicing learning and self-regulation skills and developing each student’s career and life goals (Bhat & Stevens, 2021; Cleary et al., 2017; Protivnak et al., 2016). School counselors can also provide appropriate resources based on each student’s needs. For instance, when a student does not receive academic support from parents, school counselors can help the student find alternative sources of support, such as community centers and mentoring (Hart et al., 2024; Nelson et al., 2020). Moreover, this study indicates that secondary school students’ perceptions of the helpfulness of school counseling are associated with their self-esteem, which brings about a question for school counselors about what makes students perceive school counseling as helpful. For a better understanding of student needs and evidence-based practices, school counselors can actively communicate with students, ask for their feedback, and maintain collaboration and consultation with other school mental health professionals, clinical supervisors, and university faculty. Given that school counseling support was associated with decreased negative self-esteem, rather than increased positive self-esteem, school counselors can develop programs based on more preventive and strength-based approaches that may potentially support students’ psychological well-being (Harris et al., 2026; Owens, 2022).

In addition, as another important stakeholder of collaboration and consultation, teachers can provide systematic support for students by connecting with their parents, other school personnel, and community partners (Bradley-Levine, 2018; Smith et al., 2020). Teachers can play a role in supporting students’ holistic development as part of a broader effort aimed at enhancing self-esteem, such as teaching academic skills and career development strategies (e.g., Keele et al., 2020). Lastly, school administrators can support school personnel to develop and implement curricula for students’ holistic development and well-being (Zhang, 2026). To facilitate collaboration among school professionals, administrators can promote the creation of a positive and supportive school environment. With a deeper understanding of the roles and needs of school professionals, school administrators can provide appropriate school-level support through professional development opportunities for teachers and school counselors, and parent education initiatives. Additionally, school administrators can encourage teachers to engage families in student holistic development by exercising transformational leadership to build partnerships (Jung & Sheldon, 2020).

In terms of research, first of all, researchers can refer to the findings of this study to deepen and expand the scope and design of their research. For instance, future studies can utilize longitudinal research designs to answer causal research questions, using selected instruments with appropriate reliability and validity. Moreover, researchers can recruit a nationwide sample of students, including those from schools in the Western area. Researchers can apply other research designs to examine the role of school counseling on students’ socioemotional development. For instance, they can assess students’ development depending on school counseling service availability, regardless of their helpfulness, and specifically focusing on the helpfulness of individual (or group) counseling in schools. Second, several studies noted that self-esteem development during childhood and adolescence can differ by age (Cvencek et al., 2024; Erol & Orth, 2011). Thus, examining the developmental trajectories of students’ self-esteem may help elucidate the underlying developmental mechanisms involving interactions between intrapersonal and environmental factors, specifically given that the findings of this study posed a possibility that positive self-esteem and negative self-esteem have different developmental mechanisms. In this regard, researchers can also seek a more comprehensive understanding of positive/negative self-esteem development by exploring strong potential independent variables, such as multifaceted parental involvement beyond academic support (Cetın & Taskın, 2016), teacher–student relationships (Marini et al., 2023), peer relationships (Gruenenfelder-Steiger et al., 2016), and the level of school–family–community partnerships (Bryan et al., 2020). Furthermore, the research model of this study can expand to include the consequences of positive/negative self-esteem, including emotional well-being and academic/career achievement.

## Figures and Tables

**Table 1:** Descriptive statistics and bivariate correlation coefficients.

Variable	1	2	3	4	5	6	7	8
1. Positive self-esteem	-							
2. Negative self-esteem	−0.40 ***	-						
3. Academic achievement	0.23 ***	−0.06 *	-					
4. Happiness	0.35 ***	−0.35 ***	0.15 ***	-				
5. Future readiness	0.12 ***	−0.08 **	0.08 **	0.07 **	-			
6. Parental academic support	0.30 ***	−0.13 ***	0.12 ***	0.17 ***	0.04	-		
7. School counseling support	0.08 **	−0.30 ***	−0.06 *	0.21 ***	0.04	−0.06 *	-	
8. School mental health support	0.06 *	−0.28 ***	−0.05 *	0.18 ***	0.04	0.05	0.92 ***	-
*M*	13.78	10.30	6.65	1.97	2.49	5.53	6.22	6.19
*SD*	3.75	4.51	2.13	0.56	0.85	1.93	2.88	2.92

*Note*. *N* = 1636. * *p* < 0.05; ** *p* < 0.01; *** *p* < 0.001.

**Table 2:** Participant positive self-esteem by intrapersonal and environmental factors.

	Positive Self-Esteem					
	Model 1 *B* (β)	*SE*	Model 2 *B* (β)	*SE*	Model 3 *B* (β)	*SE*
Step 1: Covariates						
Sex	−0.59 (−0.10) **	0.17	−0.39 (−0.07) **	0.16	−0.39 (−0.07) *	0.16
Race	−0.24. (−0.03)	0.23	0.01 (0.01)	0.21	0.19 (0.03)	0.21
Grade (8th/10th)	0.20 (0.05)	0.11	0.09 (0.02)	0.10	0.16 (0.04)	0.10
School region	−0.06 (−0.01)	0.13	−0.01 (−0.01)	0.12	0.08 (0.02)	0.12
School setting	0.29 (0.04)	0.19	0.31 (0.05)	0.17	0.17 (0.03)	0.17
Step 2: Intrapersonal factors						
Academic achievement			0.34 (0.19) ***	0.05	0.30 (0.17) ***	0.05
Happiness			1.92 (0.29) ***	0.18	1.70 (0.26) ***	0.18
Future readiness			0.38 (0.13) ***	0.08	0.34 (0.12) ***	0.07
Step 3: Environmental factors						
Parental academic support					0.42 (0.22) ***	0.05
School counseling support					−0.01 (−0.01)	0.04
*R*	0.12		0.42		0.47	
Adjusted *R*^2^	0.01		0.17		0.22	
Δ*R*^2^			0.16		0.05	
Δ*F*	3.75 **		79.12 ***		34.23 ***	

*Note*. *N* = 1636. * *p* < 0.05; ** *p* < 0.01; *** *p* < 0.001. Participants were secondary school students (8th or 10th grade) who had received both individual and group school counseling services. School setting was classified as city, suburban, or rural.

**Table 3:** Participant negative self-esteem by intrapersonal and environmental factors.

	Negative Self-Esteem					
	Model 1 *B* (β)	*SE*	Model 2 *B* (β)	*SE*	Model 3 *B* (β)	*SE*
Step 1: Covariates						
Sex	1.76 (0.25) ***	0.20	1.44 (0.20) ***	0.19	1.26 (0.18) ***	0.19
Race	−0.40 (−0.04)	0.27	−0.54 (−0.06)	0.25	−0.31 (−0.04)	0.25
Grade (8th/10th)	−0.37 (−0.08) **	0.13	−0.33 (−0.08) **	0.12	−0.31 (−0.07) **	0.12
School region	0.06 (0.01)	0.16	0.03 (0.01)	0.15	0.03 (0.01)	0.15
School setting	−0.28 (−0.04)	0.22	−0.25 (−0.03)	0.21	−0.26 (−0.03)	0.20
Step 2: Intrapersonal factors						
Academic achievement			−0.08 (−0.04)	0.06	−0.12 (−0.06) *	0.06
Happiness			−2.42 (−0.31) ***	0.22	−2.03 (−0.26) ***	0.22
Future readiness			−0.10 (−0.03)	0.09	−0.08 (−0.02)	0.09
Step 3: Environmental factors						
Parental academic support					−0.10 (−0.04)	0.06
School counseling support					−0.34 (−0.21) ***	0.04
*R*	0.27		0.42		0.47	
Adjusted *R*^2^	0.07		0.17		0.21	
Δ*R*^2^			0.10		0.04	
Δ*F*	18.06 ***		47.38 ***		32.69 ***	

*Note*. *N* = 1636. * *p* < 0.05; ** *p* < 0.01; *** *p* < 0.001. Participants were secondary school students (8th or 10th grade) who had received both individual and group school counseling services. School setting was classified as city, suburban, or rural.

## Data Availability

The data that support the findings of this study are publicly available from the ICPSR database: https://www.icpsr.umich.edu/ (accessed on 10 December 2025), subject to registration and data use agreement (https://doi.org/10.3886/ICPSR39445.v2).
